# Anticoagulant, Antioxidant and Antitumor Activities of Heterofucans from the Seaweed *Dictyopteris delicatula*

**DOI:** 10.3390/ijms12053352

**Published:** 2011-05-23

**Authors:** Kaline Dantas Magalhaes, Leandro Silva Costa, Gabriel Pereira Fidelis, Ruth Medeiros Oliveira, Leonardo Thiago Duarte Barreto Nobre, Nednaldo Dantas-Santos, Rafael Barros Gomes Camara, Ivan Rui Lopes Albuquerque, Sara Lima Cordeiro, Diego Araujo Sabry, Mariana Santana Santos Pereira Costa, Luciana Guimaraes Alves, Hugo Alexandre Oliveira Rocha

**Affiliations:** 1 Laboratory of Biotechnology of Natural Polymers (BIOPOL), Department of Biochemistry, Federal University of Rio Grande do Norte (UFRN), 59020-100 Natal-RN, Brazil; E-Mails: kdmbio@yahoo.com.br (K.D.M.); leandro-silva-costa@hotmail.com (L.S.C.); gabrielfideliss@gmail.com (G.P.F.); rmo_85@hotmail.com (R.M.O.); leo_dnobre@yahoo.com.br (L.T.D.B.N.); nednaldod@hotmail.com (N.D.-S.); rafael_bgc@yahoo.com.br (R.B.G.C.); ivan.rui@click21.com.br (I.R.L.A.); sara-cordeiro@hotmail.com (S.L.C.); popoh_diego@hotmail.com (D.A.S.); marispc_bio@yahoo.com.br (M.S.S.P.C.); lucianagalves@hotmail.com (L.G.A.); 2 Health Post-Graduate Program, Federal University of Rio Grande do Norte (UFRN), 59020-100 Natal-RN, Brazil; 3 Federal Institute of Education, Science and Technology of Rio Grande do Norte (IFRN), 59200-000 Santa Cruz-RN, Brazil

**Keywords:** fucoidan, brown seaweed, biological activities, sulfated polysaccharides

## Abstract

In the present study, six families of sulfated polysaccharides were obtained from seaweed *Dictyopteris delicatula* by proteolytic digestion, followed by acetone fractionation and molecular sieving on Sephadex G-100. Chemical analyses demonstrated that all polysaccharides contain heterofucans composed mainly of fucose, xylose, glucose, galactose, uronic acid, and sulfate. The fucans F0.5v and F0.7v at 1.0 mg/mL showed high ferric chelating activity (∼45%), whereas fucans F1.3v (0.5 mg/mL) showed considerable reducing power, about 53.2% of the activity of vitamin C. The fucan F1.5v presented the most prominent anticoagulant activity. The best antiproliferative activity was found with fucans F1.3v and F0.7v. However, F1.3v activity was much higher than F0.7v inhibiting almost 100% of HeLa cell proliferation. These fucans have been selected for further studies on structural characterization as well as *in vivo* experiments, which are already in progress.

## 1. Introduction

Marine seaweeds (green, red and brown) are the most abundant source of non-mammalian anticoagulant sulfated polysaccharides in nature. Some structural similarities between sulfated seaweed polysaccharides and heparin led several groups to study algal polysaccharides as antithrombotic [1], anti-adhesive [2], antitumoral [3], antiviral [4], anticoagulant, antioxidant, proangiogenic, anti-inflammatory, anthelmintic compounds [5,6]. The well known sulfated polysaccharides from red seaweed are homogalactans [7] and from brown algae are α-l-fucose-containing sulfated homo and heteropolysaccharides [6] called fucan and fucoidan, respectively. On the other hand, green algae synthesize several kinds of sulfated homo and heteropolysaccharides [6].

The Brazilian’s northeast coast has a great variety of seaweeds. However, few pharmacognostical and pharmacological investigations about these seaweeds are carried out to identify new drugs or to find new structures for the development of novel therapeutic agents for the treatment of human diseases such as cancer and infectious diseases [8].

In Brazil, the brown seaweed *Dictyopteris delicatula* is common along the northeast coast. Recently, we obtained a polysaccharide-rich extract from *D. delicatula*, which exhibited assorted biological activities, including anticoagulant, antiproliferative and antioxidant activities [9]. However, the biological activities of the purified sulfated polysaccharides from *D. delicatula* have not been investigated.

On the basis of these considerations, the purpose of the present study was to obtain sulfated polysaccharides from *D. delicatula* and to evaluate their anticoagulant, antioxidant and antiproliferative activities *in vitro*. Our results show several noteworthy differences in the activities of polysaccharides from *D. delicatula*, which are likely connected to differences in the chemical structure of these compounds. This work was directed to the selection of the most active sulfated polysaccharide samples to be studied further as potential novel drugs for thrombosis, antitumor and/or antioxidant therapy.

## 2. Results and Discussion

### 2.1. Chemical Analysis

Using a methodology that combined proteolysis and acetone precipitation we obtained six polysaccharides preparations from brown seaweed *D. delicatula*. The addition of increasing volumes of acetone gradually decreased the dielectric constant of water, thereby promoting different sulfated polysaccharide precipitation rates. Acetone separates polysaccharides by the way in which the charges of these polymers interact with water. Thus, those that interact more with water are the last to precipitate. Each fraction was subjected to molecular sieving on Sephadex G-100, monitored for total sugar and metachromasia. Each fraction had a single peak, which indicates that each fraction is composed of a unique population of sulfated polysaccharide (Data not shown). The tubes containing the sulfated polysaccharides were pooled and lyophilized, and the sulfated polysaccharides were also named F0.5v; F0.7v; F1.0v; F1.3v; F1.5v and F2.0v respectively.

The chemical analysis of sulfated polysaccharides is summarized in Table 1; sulfate and fucose were found in all polysaccharides. Moreover, other neutral monosaccharides were also found as components of these polymers indicating that that *D. delicatula* synthesizes at least six families of sulfated heterofucans.

The heterofucan F1.0v was the polysaccharide with the higher percentage of sulfate (19.0%) in comparison to other heterofucans. In addition, when the sulfate/total sugar ratio was determinate (Table 1) F1.0v showed the highest ratio, whereas F0.5v presented the smallest ratio. All the polysaccharides showed low protein contamination, which ranged from 0.1 (F0.5v, F1.3v and F1.5v) to 0.7% (F2.0v); this is due to the use of the proteolytic enzyme during the method of polysaccharide extraction.

The monosaccharide composition of sulfated polysaccharides is also shown in Table 1. All fractions showed the same constitution on monosaccharides: fucose, galactose, glucose, mannose, xylose and glucuronic acid. The data showed galactose as the main sugar present in the all of the heterofucans, but the amount of this sugar is different in each polymer. In addition, there are differences among the relative proportions of glucuronic acid and xylose observed in different polysaccharides; they are found in greater amounts in F0.7v; F1.5v and F2.0v. On the other hand, the proportion of mannose and glucose did not show higher differences among the heterofucans. Thus, it is clear that the relative amounts of these sugars vary according to the fucan extracted.

Most brown seaweeds synthesize sulfated polysaccharides consisting mainly of sulfated l-fucose with fucose content about 34–44%. They may as well contain small portions of galactose, mannose, glucose, xylose and glucuronic acid [5]. However, there are also heterofucans with only minor fucose components. In these polysaccharides, other monosaccharides like glucuronic acid [10] exceed the fucose content.

It is well known that several brown seaweeds produced only one kind fucan, this not a rule and several brown seaweeds synthesize more than one kind of fucan. Using the same method described in this paper we have extracted three fucans from *Dictyota mertensii* [11] and *Spatoglossum schröederi* [10] as well as five fucans from *Dictyota menstrualis* [12] and *Padina gymnospora* [13]. In addition, other studies have shown brown seaweed synthesizing more than one fucan, including *Adenocystis utricularis* [14], *Analipus japonicus* [15], *Ascophylum nodosum* [16,17], *Sargassum vulgare* [11], *Padina pavonia* [18], *Sargassum stenophylum* [19]. Heterofucans from brown seaweed have been described since 1950 [20] and in some cases galactose was reported to be a major component [21,12]. Because they have not been frequently described, we decided to analyze the some biological activities of these heterofucans from *D. delicatula*.

### 2.2. Anticoagulant Activity by APTT and PT Assays

**Figure 1.**
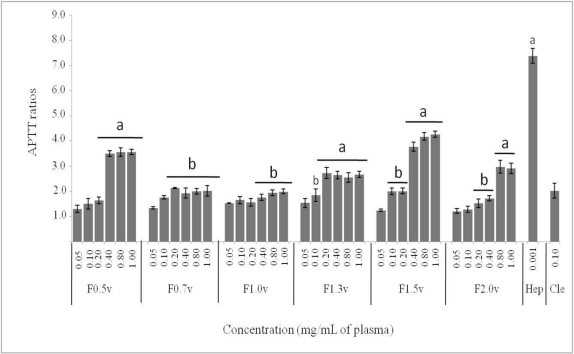
Anticoagulant activity of sulfated polysaccharides from *Dictyopteris delicatula.* Results were expressed as ratios obtained by dividing the clotting time achieved with the anticoagulant by the time achieved with the control. Hep—Heparin; Cle—Clexane^®^ (Enoxaparin). Each value is the mean ± SD of three determinations (*n* = 6). ^a^ indicates a significant difference when compared with Clexane^®^ control; ^b^ indicates a similar APTT ratio when compared with Clexane^®^ control.

The anticoagulant properties of the heterofucans were evaluated using PT and APTT assays. No clotting inhibition was observed in PT test of any of the samples at the concentrations assayed (data not shown). On the other hand, APTT were prolonged by all the polysaccharides, in a dose-dependent manner, with regards to the control (Figure 1). The heterofucans F0.7v and F1.0v showed low anticoagulant activity while F1.5v presented the most prominent anticoagulant activity with 3.8 of APTT ratio with only 0.40 mg/mL of plasma. When compared to Clexane^®^, a low molecular weight heparin, at same concentration F1.5v presented similar anticoagulant activity (*p* < 0.05).

Fucans have a wide variety of biological activities, but their potent anticoagulant action is by far the most widely studied. The heterofucans from *D. delicatula* exhibited anticoagulant activity by APTT test only, which suggested that the sulfated polysaccharide extracted from *D. delicatula* inhibited both the intrinsic and/or common pathways of coagulation. In addition, none of the fucans from *D. delicatula* affected a PT test, which indicates that the extrinsic pathway of coagulation would not be inhibited or, at least, the high kinetic of the assay would not allow the detection of the anticoagulant activity of these polymers. All the fucans from *D. delicatula* showed anticoagulant activity in different levels, however there was no correlation between total sulfate content and the APTT test (R^2^ = 0.190). Thus, the heterofucan F1.5v was the most potent anticoagulant compound, followed by F0.5v and F2.0v. In addition, F1.5v showed anticoagulant activity similar to Clexane^®^, an anticoagulant commercial drug of reference. Our data are in agreement with several works that clearly show that the anticoagulant effect of fucans was stereo-specific and not merely a consequence of their charge density or sulfate content [22]. The position of sulfate groups on sugar residues is also very important for the anticoagulant activity of fucan. The activity relates to the concentrations of C-2 sulfate and C-2,3 disulfate [16], moreover, Silva and colleges reported that 3–*O*–sulfation at C-3 of 4-α-l-fucose-1→units was responsible for the anticoagulant activity of heterofucans from *Padina gymnospora* [13]. F1.5v has been selected for further bio-guided fractionation and isolation of active fractions containing potent anticoagulant fucans which will be further submitted to structural analysis in order to identify the structural features responsible for their anticoagulant activity.

### 2.3. Antioxidant Activity

Antioxidant activity was evaluated in different assays: scavenging hydroxyl and superoxide radicals, power reducing and ferrous chelating.

**Figure 2.**
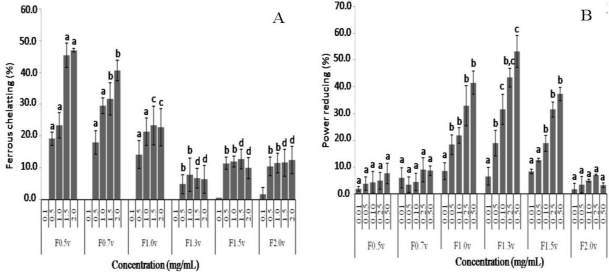
Antioxidant activity of sulfated polysaccharides from *Dictyopteris delicatula.* (**A**) Chelating effect; (**B**) Power reducing assay. Each value is the mean ± SD of three determinations. ^a,b,c,d^ Distinct letters indicates a significant difference between sulfated polysaccharides when compared at each concentration (*p* < 0.05).

Antioxidants inhibit interaction between metal and lipid through formation of insoluble metal complexes with ferrous ion or generation of steric hindrance. The iron-chelating capacity test measures the ability of antioxidants to compete with ferrozine in chelating ferrous ion. Activity is measured as the decrease in absorbance of the red Fe^2+^/ferrozine complex. The plot of iron-chelating capacity as a function of sample concentration is shown in Figure 2A. The results revealed that heterofucan F1.3v did not statistically exhibit significant differences (*p* > 0.05) in ferrous chelating capacity compared with negative control (saline, data not shown), while F1.5v and F2.0v showed very low activity. On the other hand, the heterofucans F0.5v, F0.7v and F1.0v presented a dose-dependent chelating capacity. The most active compound was F0.5v with 45.5% of ferrous chelating at 1.5 mg/mL. This activity was only 1.8 times lower than EDTA activity at the same concentration under the same experimental condition (Data not shown). The purification process did not increase the chelating effect of the heterofucans compared to the chelating effect of sulfated polysaccharide-rich extract from *D. delicatula* [9]. However, ferrous ions are considered to be the most effective pro-oxidants present in food systems [23]. Thus, the metal-chelating property of these heterofucans, mainly F0.5v and F0.7v, showed that they might be applied in adsorption, metal ions separation or wastewater treatment and antioxidant therapy.

The reducing power assay was expressed as percentage activity of ascorbic acid control at 0.1 mg/mL and the data are shown in Figure 2B. Here, the heterofucans F0.5v, F0.7v and F2.0v showed extremely low activity when compared to the activity of vitamin C (0.1 mg/mL). Furthermore, fucans F1.0v, F1.3v and F1.5v showed considerable reducing power, especially F1.3v, which showed 53.2% of the activity of vitamin C at 0.5 mg/mL. It has been previously reported that there was a direct correlation between antioxidant activities and reducing power of polysaccharide. The reducing activities were usually related to the development of reductones. Reductones were reported to be terminators of free radical chain reactions by donating a hydrogen atom. In most cases, irrespective of the stage in the oxidative chain in which the antioxidant action is assessed, most nonenzymatic antioxidative activity is mediated by redox reactions [24]. Thus, several workers have reported that the antioxidant activity was concomitant with the reducing power. Zhang *et al*. [25] showed that acetylated polysaccharides with high donating-hydrogen abilities showed excellent reducing power, and in sulfated polysaccharides, the presence of the sulfate groups lead to the diminution of hydroxyl groups, which resulted in the descent of the reducing power. Our data showed that the most active fucan (F1.3v) is the fucan that has the smallest sulfate/total sugar ratio. Thus, our data indicates that the reducing power activity of the *D. delicatula* fucans studied here depends on the spatial patterns of sulfate groups, and it is unlikely to be merely a charge density effect.

Superoxide anion scavenging results in the presence of the heterofucans or commercial antioxidant are exhibited in Table 2. In this assay, the fucans F1.0v, F1.3v and F2.0v did not show superoxide scavenging activity, while F0.5v and F0.7v presented very low superoxide scavenging with 5.7 and 4.8% at 0.5 mg/mL, respectively. Moreover, F1.5v was the unique polysaccharide with moderate superoxide radical scavenging activity (13.4% of scavenging at 0.5 mg/mL). This value is 2.4-fold less than the superoxide activity of a rich-sulfated polysaccharide extract from *D. delicatula* obtained in previous study [9]. This suggests that sulfated polysaccharides of *D. delicatula* may act synergistically to promote a more efficient superoxide radical scavenging.

We determined the hydroxyl radical scavenging activity of the heterofucans of *D. delicatula*. Table 2 depicts the results obtained for the inhibition of hydroxyl radical formation. Only the sulfated polysaccharides F0.5v, F0.7v and F1.0v have shown activity in hydroxyl radical scavenging. However, these polysaccharides showed moderate activity with scavenging activity of 14.4%, 15.6% and 17.0%, respectively, at 0.5 mg/mL. For this assay, gallic acid showed 93.7% radical scavenging effect at 0.5 mg/mL concentration.

In this work, all the polysaccharides from *D. delicatula* were poor effective hydroxyl radical scavengers, which is unsurprising, since the most of the sulfated polysaccharides reported in the literature have a weak hydroxyl radical scavenger activity [22]. However, heterofucans from *Padina gymnospora* [26], which have similar monosaccharide composition of fucans from *D. delicatula*, as well as, homofucans from *Fucus vesiculosus* and *Laminaria japonica* [26,27] have high activity as hydroxyl radical scavengers.

The results reported here are very interesting, since they indicate that brown seaweed *D. delicatula* synthesizes heterofucans with different antioxidant mechanisms. Although their antioxidant potential was not significantly high in some assays, the sum of individual effects of these polymers should be used as a defense strategy of this seaweed. This would supply a more efficient defense against free radicals.

### 2.4. Antiproliferative Activity

**Figure 3.**
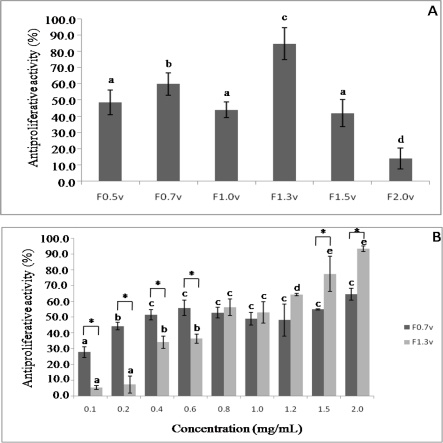
Influence of sulfated polysaccharides from *Dictyopteris delicatula* on inhibition of cell proliferation of HeLa cells after 72 h incubation: (**A**) Antiproliferative activity of sulfated polysaccharides at concentration of 2.0 mg/mL; (**B**) Dose-dependent effect of sulfated polysaccharides. Each value is the mean ± SD of seven determinations. Different letters indicates a significant difference between concentrations of individual sulfated polysaccharides (*p* < 0.05); the asterisk indicates a significant difference between different sulfated polysaccharides at same concentration (*p* < 0.05).

The viability of HeLa cells treated with sulfated polysaccharides for 24 h was determined using a colorimetric MTT-based assay (Figure 3). Initially, the antiproliferative activity of sulfated polysaccharides was evaluated in a single concentration, 2.0 mg/mL. F2.0v showed the lowest activity inhibiting only 14.1% of cell viability. The best antiproliferative activity was found with F1.3v and F0.7v that inhibit 91.8% and 60.0% of HeLa cells proliferation, respectively (Figure 3A). Thus, the antiproliferative activity of these fucans was evaluated at different concentrations.

Figure 3B demonstrates that this effect of fucan F0.7v is a dose-dependent effect, reaching the saturation around 0.4 mg/mL. In addition, it showed higher antiproliferative activity than F1.3v in low concentration (from 0.1 to 0.6 mg/mL). The heterofucan F1.3v also showed a dose-dependent antiproliferative activity, reaching the saturation around 2.0 mg/mL. However, its activity was much higher than F0.7v inhibiting almost 100 % of HeLa cell proliferation.

Earlier, we demonstrated a polysaccharide-rich extract from *D. delicatula* inhibiting Hela proliferation in a dose-dependent manner, reaching the saturation around 2.0 mg/mL (∼61%) [9]. When we purified the fucans from this seaweed, it was clear that the fucan F1.3v was the most responsible for antiproliferative activity of the polysaccharide-rich extract from *D. delicatula*, since this fucan inhibited almost 100% HeLa proliferation. There have been a number of studies on the antitumor activity of fucan *in vivo* and *in vitro* [5,6,22,28]. Although the precise mechanisms underlying this activity remain to be determined, a few possibilities have been proposed. Fucan is speculated to act by inhibiting tumor angiogenesis modulating host immune systems [22], arresting the cell cycle, and/or inducing apoptosis [29]. From the experiment carried out here it was clear that the mechanism of action of F1.3v is directly on the HeLa cell. Further studies will clarify the antiproliferative mechanism of fucan F1.3v.

## 3. Experimental Section

### 3.1. Materials

Arabinose, mannose, galactose, xylose, fucose, glucuronic acid and unfractionated heparin were obtained from Sigma (St. Louis, MO, USA). Acetone and sulfuric acid were obtained from Merck (Darmstadt, Germany). Clexane^®^ (enoxaparin) was purchased from Aventis (São Paulo, Brazil).

### 3.2. Extraction of Polysaccharides

The marine alga *Dictyopteris delicatula* Lamouroux was collected in the sub littoral of Natal, RN, Brazil. The extraction of sulfated polysaccharide followed the procedure described by Rocha *et al*. [30]. Immediately after collection, the seaweed was dried at 50 °C under ventilation and ground in a blender. Next, it was treated with acetone to eliminate lipids and pigments. One hundred grams of defatted, dried, and powdered alga were suspended in 500 mL of 0.25 M NaCl, and the pH was adjusted to 8.0 with NaOH. Twenty milligrams of maxatase, an alkaline protease from *Esporobacillus* (Biobras, MG, Brazil), was added to the mixture for proteolytic digestion. After 18 h of incubation at 60 °C under agitation, the mixture was filtered through cheesecloth. The filtrate, which is the *D. delicatula’* polysaccharide-rich extract, was fractionated by acetone precipitation as follows: 0.5 volumes of ice-cold acetone was added to the solution under gentle agitation and maintained at 4 °C for 24 h. The precipitate formed was collected by centrifugation (10.000 × g, 20 min), vacuum dried, resuspended in distilled water, and analyzed. The operation was repeated by adding 0.7, 1.0, 1.3, 1.5 and 2.0 volumes of acetone to the supernatant. These fractions were further purified by molecular sieving in Sephadex G-100 (140 × 2.6 cm). About 200 mg of each fraction, dissolved in 2 mL of water, was applied to the column and eluted with a solution of 0.2 M acetic acid and 0.15 M NaCl, and fractions of 1 mL were collected and assayed by the phenol/H_2_SO_4_ reaction [31] and by metachromatic assay using 1,9-dimethylmethylene blue [32]. The fractions containing the sulfated polysaccharides were pooled, dialyzed against distilled water, and lyophilized.

### 3.3. Chemical Analysis and Monosaccharide Composition

Total sugars were estimated by the phenol-H_2_SO_4_ reaction [31] using l-fucose as standard. Sulfate content was determined according to the gelatin-barium method [33], using sodium sulfate (1 mg/mL) as standard and after acid hydrolysis of the polysaccharides (4 M HCl, 100 °C, 6 h). Protein content was measured using Spector’s method [34].

The polysaccharides were hydrolyzed with 0.5, 1, 2, and 4 M of HCl, respectively, for various lengths of time (0.5, 1, 2 and 4 h) at 100 °C. Reducing sugars were determined using the Somogyi-Nelson method [35]. After acid hydrolysis, sugar composition was determined by a LaChrom Elite^®^ HPLC system from VWR-Hitachi with a refractive index detector (RI detector model L-2490). A LichroCART^®^ 250-4 column (250 mm × 40 mm) packed with Lichrospher^®^ 100 NH_2_ (5 μm) was coupled to the system. The sample mass used was 0.2 mg and analysis time was 25 min. The following sugars were analyzed as references: arabinose, fructose, fucose, galactose, glucose, glucosamine, glucuronic acid, mannose, and xylose. The amount of uronic acid was determinate as described by Leite *et al*. [10].

### 3.4. Anticoagulant Activity

All the Protrombin Time (PT) and Activated Partial Thromboplastin Time (APTT) coagulation assays were performed with a coagulometer as described earlier [12] and measured using citrate-treated normal human plasma. All assays were performed in duplicate and repeated at least three times on different days (*n* = 6). The results were expressed as APTT ratio, which was determined as follows: APTT control time/APTT sample time.

### 3.5. Antioxidant Activity

To analyze the antioxidant activity of the sulfated polysaccharides obtained, three assays were performed: hydroxyl radical scavenging, superoxide radical scavenging, and ferric chelating, as described previously [9].

#### 3.5.1. Hydroxyl Radical Scavenging Activity Assay

The scavenging activity of seaweed sulfated polysaccharides against the hydroxyl radical was investigated using Fenton’s reaction (Fe^2+^ + H_2_O_2_ → Fe^3+^ + OH^−^ + OH^·^). These results were expressed as an inhibition rate. Hydroxyl radicals were generated using a modified method [17] in 3 mL sodium phosphate buffer (150 mM, pH 7.4), which contained 10 mM FeSO_4_ · 7H_2_O, 10 mM EDTA, 2 mM sodium salicylate, 30% H_2_O_2_ (200 μL) and varying sulfated polysaccharide concentration. In the control, a sodium phosphate buffer replaced H_2_O_2_. The solutions were incubated at 37 °C for 1 h, and the presence of the hydroxyl radical was detected by monitoring absorbance at 510 nm.

#### 3.5.2. Superoxide Radical Scavenging Activity Assay

The assay was based on the capacity of sulfated polysaccharides to inhibit the photochemical reduction of nitroblue tetrazolium (NBT) in the riboflavin–light–NBT system. Each 3 mL reaction mixture contained 50 mM phosphate buffer (pH 7.8), 13 mM methionine, 2 μM riboflavin, 100 μM EDTA, NBT (75 μM), and 1 mL sample solution. The production of blue formazan was followed by monitoring the increase in absorbance at 560 nm after 10 min illumination from a fluorescent lamp. The entire reaction assembly was enclosed in a box lined with aluminum foil. Identical tubes with the reaction mixture were kept in the dark and served as blanks.

#### 3.5.3. Ferric Chelating

The ferrous ion chelating ability of samples was investigated according to previous studies [9]. Briefly, the reaction mixture, containing samples of FeCl_2_ (0.05 mL, 2 mM) and ferrozine (0.2 mL, 5 mM), was well shaken and incubated for 10 min at room temperature. The absorbance of the mixture was measured at 562 nm against a blank.

### 3.6. Cell Proliferation Studies

HeLa cells were grown in 75 cm^2^ flasks in DMEM medium. Cells were seeded into 96-well plates at a density of 5 × 10^3^ cell/well and allowed to attach overnight in 300 μL medium incubated at 37 °C, 5% CO_2_. The sulfated polysaccharides fractions were added at a final concentration of 0.01; 0.1; 1.0 and 2.0 mg/mL, for 72 h at 37 °C and 5% CO_2_. After incubation, traces of sulfated polysaccharides fractions were removed by washing the cells twice with 200 μL PBS and applying 100 μL of fresh medium plus and 10 μL of 12 mM MTT dissolved in PBS to determine the effects of the algal sulfated polysaccharides on cell proliferation. Cells were then incubated for 4 h at 37 °C, 5% CO_2_. In order to solubilize the product of MTT cleavage, 100 μL of isopropanol containing 0.04 M HCl was added to each well and thoroughly mixed using a multichannel pipettor. Within 1 h of HCl-isopropanol addition, the absorbance at 570 nm was read using a Multiskan Ascent Microplate Reader (Thermo Labsystems, Franklin, MA). The percent inhibition of cell proliferation was calculated as follows:

$$\%\text{Inhibition}=\frac{\text{Abs}.\;570\;\text{nm}\;\text{control}-\text{Abs}.\;570\;\text{nm}\;\text{sample}}{\text{Abs}.\;570\;\text{nm}\;\text{control}}\times 100$$

Each concentration of the respective sulfated polysaccharide was assayed in seven-fold.

### 3.7. Statistical Analysis

All data were expressed as mean ± standard deviation. Statistical analysis was done by one-way ANOVA using the SIGMAStat version 2.01 computer software. Student-Newmans-Keuls post-tests were performed for multiple group comparison. In all cases statistical significance was set at *p* < 0.05.

## 4. Conclusions

In conclusion, we have extracted six heterofucans from the brown seaweed *D. delicatula* which showed anticoagulant, antioxidant and antiproliferative activities at different levels of activity. However, it has become clear that at least some of these activities are not merely an effect of high charge density but have distinct structural specificities. Future conformational studies of well-defined fucan structures should lead to a better understanding of the biological properties of these fucans.

## Figures and Tables

**Table 1.** Chemical composition of sulfated polysaccharides extracted from *Dictyopteris delicatula.*

**Fucans**	**Total sugar (%)**	**Sulfate (%)**	**Sulfate/Total sugar (%/%)**	**Proteins (%)**	**Molar ratio**					
					**Fuc**	**Gal**	**Glc**	**Man**	**Xyl**	**Gluc A**
F0.5v	89.1	14.1	0.16	0.1	1.0	2.0	0.3	0.5	0.7	0.9
F0.7v	70.0	17.8	0.25	0.3	1.0	3.0	0.4	0.6	1.5	2.2
F1.0v	65.0	19.0	0.29	0.5	1.0	1.9	0.2	0.5	0.8	1.5
F1.3v	68.2	14.5	0.21	0.1	1.0	2.4	0.3	0.2	0.6	0.5
F1.5v	61.3	15.4	0.25	0.1	1.0	1.6	0.2	0.4	1.3	1.9
F2.0v	64.3	16.0	0.25	0.7	1.0	1.8	0.2	0.4	1.6	2.2

Fuc—fucose; Gal—galactose; Glc—glucose; Man—mannose; Xyl—xylose; Gluc A—glucuronic acid.

**Table 2.** Hydroxyl and superoxide radical scavenging activity of sulfated polyssacharides from *Dictyopteris delicatula*.

**Sulfated polysaccharides**	**Concentration (mg/mL)**	**Inhibition (%)**	
		OH^·^	O_2_^−^
F0.5v	0.05	6.1 ± 0.7	2.8 ± 2.1
	0.1	9.6 ± 0.4	4.9 ± 3.1
	0.25	11.0 ± 0.9	5.1 ± 3.4
	0.5	14.4 ± 2.1	5.7 ± 4.0

F0.7v	0.05	0.5 ± 0.7	3.9 ± 0.3
	0.1	12.4 ± 3.5	6.1 ± 0.9
	0.25	13.4 ± 4.7	6.7 ± 3.1
	0.5	15.6 ± 3.8	4.8 ± 2.4

F1.0v	0.05	6.6 ± 3.1	0 ± 0
	0.1	12.7 ± 2.9	0 ± 0
	0.25	16.9 ± 2.2	0 ± 0
	0.5	17.0 ± 3.4	0 ± 0

F1.3v	0.05	0 ± 0	0 ± 0
	0.1	0 ± 0	0 ± 0
	0.25	0 ± 0	0 ± 0
	0.5	0 ± 0	0 ± 0

F1.5v	0.05	0 ± 0	3.9 ± 2.1
	0.1	0 ± 0	4.9 ± 3.1
	0.25	0 ± 0	9.6 ± 3.4
	0.5	0 ± 0	13.4 ± 2.1

F2.0v	0.05	0 ± 0	0 ± 0
	0.1	0 ± 0	0 ± 0
	0.25	0 ± 0	0 ± 0
	0.5	0 ± 0	0 ± 0

	0.1	43.6 ± 2.4	41.8 ± 4.7
	0.25	64.3 ± 3.0	72.1 ± 2.9
	0.5	93.7 ± 3.7	86.3 ± 3.1
